# Effects of Grass Inter-Planting on Soil Nutrients, Enzyme Activity, and Bacterial Community Diversity in an Apple Orchard

**DOI:** 10.3389/fpls.2022.901143

**Published:** 2022-06-27

**Authors:** Tengfei Li, Yingying Wang, Muhammad Kamran, Xinyi Chen, Hua Tan, Mingxiu Long

**Affiliations:** ^1^College of Grassland Agriculture, Northwest A&F University, Yangling, China; ^2^State Key Laboratory of Grassland Agro-Ecosystems, Key Laboratory of Grassland Livestock Industry Innovation, Ministry of Agriculture and Rural Affairs, College of Pastoral Agriculture Science and Technology, Lanzhou University, Lanzhou, China

**Keywords:** bacteria, community diversity, enzyme activity, grass inter-planting, soil nutrients

## Abstract

The orchard inter-planting pattern is being widely used in many countries of the world, but it is relatively new in China. This study evaluated the interrow mono- and mixed-planting of *Lolium perenne* (Lp) and *Medicago sativa* (Ms) in orchards on soil nutrient, enzyme activity, and bacterial community diversity in 0–10, 10–20, and 20–40 cm soil layers. The clean tillage orchard was used as control (CK) treatment. Compared with CK, Lp and Lp + Ms. significantly increased the contents of soil organic matter (OM), total nitrogen (TN), and alkali-hydrolyzable nitrogen (AN) in 0–20-cm soil layer, and up-regulated the activities of urease (URE) and alkaline phosphatase (ALP). The Lp treatment significantly increased the relative abundance of *Gemmatimonadetes* and *Planctomycetes* in the 0-10-cm soil layer. Besides, cover crops significantly increased the abundance of *Actinobacteria*, *Gemmatimonadetes*, and *Chloroflexi* in the 10–20-cm soil layer and that of *Gemmatimonadetes* and *Chloroflexi* in the 20–40 cm soil layer. The redundancy analysis (RDA) showed significant positive correlations of *Actinobacteria* with ALP, OM and TN and that of *Bacteroidetes* with available potassium (AK), and *Proteobacteria* with available phosphorus (AP). Overall, the grass inter-planting improved the soil nutrients, enzymes activities, and bacterial community composition of the soil. Based on these results, inter-planting perennial ryegrass in the apple orchards is a suitable grass-orchard inter-planting strategy in Weibei, Shaanxi Province of China.

## Introduction

Apple orchards cover a planted area of more than 10 million acres and provides an output of more than 11 million tons in Shaanxi province of China (Peng et al., 2017). In Shaanxi, Weibei is one of the major apple producing areas, with abundant sunshine and mean annual temperature of 12–14°C. However, orchard management based on traditional tillage has resulted in a series of problems, such as decreased soil fertility, microbial communities (Zhou et al., 2014, 2015), and apple yield, and quality (Qian et al., 2019). Therefore, the sustainable development of apple orchards in this region is facing severe challenges.

Grass inter-planting has been proven to be an effective system for orchard management (De Baets et al., 2011; Nieto et al., 2013; Qian et al., 2015; Vicente-Vicente et al., 2017). Previous studies have demonstrated an increase in soil organic matter content (Marquez-Garcia et al., 2013; Nieto et al., 2013; Chen et al., 2014; Vicente-Vicente et al., 2017) and water infiltration and a decrease in soil erosion (Delpuech and Metay, 2018) and evaporation (Ling et al., 2017) with grass inter-planting. The grass inter-planting system increases the total nitrogen, available phosphorus, and available potassium contents of the soil, especially in the top soil (Hoagland et al., 2008), and maintains the soil nitrogen content (Verzeaux et al., 2016). It also promotes the activity of soil enzymes, such as β-glucosidase, β-xylosidase, and cellobiohydrolase, involved in plant polysaccharide (cellulose and hemicellulose) hydrolysis (Zheng et al., 2018).

Soil bacterial communities participate in various processes such as carbon and nitrogen cycling, organic matter decomposition, soil agglomeration and humus formation, and may form symbiotic and parasitic relationships with plants (Powlson et al., 2001). Studies have shown that inter-planting grass significantly influences soil bacterial structure, including the community diversity (Qian et al., 2015), and soil bacterial function, such as the carbon metabolism-related activity. However, different types of grass inter-planting have different effects on the soil bacterial communities’ structure and function. Therefore, understanding the effects of sowing forages in an apple orchard on soil nutrients and bacterial community diversity is of great theoretical and practical significance to orchard management in Weibei.

In this study, two different pastures were grown in the apple orchard of Weibei to analyze the effects of grass inter-planting on soil nutrients, enzyme activities, and microbial community diversity in the plow layer. This study will provide a theoretical and technical basis for selecting suitable grass-species and sowing method for orchard grass management.

## Materials and Methods

### Experiment Site Location

The experiment was conducted in Gaoyang Town of Pucheng County (108°20′17′E, 34°26′50′N) in the Weibei area of Shaanxi Province. The area has a temperate continental monsoon climate with distinct seasons and plenty of sunshine. The annual average temperature is 13.3°C, precipitation is 524.1 mm, sunshine duration is approx. 2277.5 h, and the frost-free period is 218 days. Changfu 2 (belongs to long branch type, with large crown, upright tree posture and strong growth vigor), Jinshiji (compact crown, easy to flower, early fruit, and high yield), Ruiyang (early fruit, high yield, strong resistance, easy cultivation, and good storage), and Ruixue (regular fruit shape, good quality, durable storage, high yield, with short branch characteristics) are the major apple varieties grown in the region, at a plant spacing of 2 m and a row spacing of 4 m. The orchard was built in 2016, with zero or only natural weeds between the rows before the experiment.

### Experimental Design

A field experiment was carried out in September 2018 using two local dominant grass species, alfalfa (*Medicago sativa* L.) and perennial ryegrass (*Lolium perenne* L.). Four treatments were established: alfalfa inter-planting (Ms), perennial ryegrass inter-planting (Lp), alfalfa and perennial ryegrass mixed inter-planting (Lp + Ms), and clearing tillage (control, CK). The treatments were organized in a randomized complete design, with four replications per treatment. The size of each treatment plot was 200 m^2^ (4 m × 50 m). A 1 m long buffer zone was maintained between the adjacent plots to avoid competition between the grass species and the apple trees.

### Soil Sampling

Soil samples were collected randomly by five sampling points within each plot in July 2020. Samples were collected from the 0–10, 10–20, and 20–40 cm soil layers using a soil drill. The samples from each plot were mixed well and divided into three parts. The first part was transported in an ice box and stored in a freezer at −20°C in the laboratory to determine the diversity of microbial communities. The second part was filtered through a 2 mm soil sieve to determine the soil enzyme activity. The third part was air-dried and filtered through a 2 mm soil sieve to measure the soil nutrient content.

### Soil Analysis

#### Determination of Soil Nutrient Content

Total nitrogen (TN) was estimated by the Kjeldahl method (Bao, 2000). Total phosphorus (TP) and available phosphorus (AP) were estimated by molybdenum-antimony anticolorimetry (Cheng et al., 2021). Organic matter (OM) content was assayed following the dichromate oxidation method (Bremner and Jenkinson, 1960). Soil pH was measured following the electrode potential method (1:2.5, soil:water; Cheng et al., 2021). Available potassium (AK) was estimated by sodium tetraphenylborate turbidity and alkali-hydrolyzed nitrogen (AN) by the alkali N-proliferation method (Lijuan et al., 2020).

#### Determination of Soil Enzyme Activities

Catalase (CAT), Urease (URE) and Alkaline Phosphatase (ALP) activity refer to the test method of Jing et al. (2020). Sucrase (SUA) activity was determined by 3,5-dinitrosalicylic acid colorimetry (Guan, 1986). β-Glucosidase (BG), cellobiohydrolase (CBH), and β-N-acetylglucosaminidase (NAG) activities were analyzed following the microplate fluorescence method, nitrophenol colorimetric method, and multifunctional microplate reader method, respectively (Trap et al., 2012).

#### Molecular Identification of Soil Bacteria by 16 s rDNA Sequencing

The total DNA was extracted using an E.Z.N.A.^®^Soil DNA Kit following the manufacturer’s instruction. The quality of the extracted DNA was analyzed by agarose gel electrophoresis, and quantity was measured by UV spectrophotometer. The V3–V4 variable region of the 16SrDNA was amplified by PCR using 341F (5′-CCTACGGGNGGCWGCAG-3′) and 805R (5′-GACTACHVGGGTATCTAATCC-3′) primers (Verzeaux et al., 2016). The PCR product’s quality was confirmed by agarose gel electrophoresis (2%; w/v), and the target fragment was recovered using the AxyPrep PCR Cleanup Kit. The PCR product was further purified using AMPure XT beads (Beckman Coulter Genomics, Danvers, MA, United States of America) and quantified on a Qubit fluorometer (Invitrogen, United States of America). The size and quantity of the amplicon library were evaluated on the Agilent 2100 Bioanalyzer (Agilent, United States of America) and using Illumina (Kapa Biosciences, Woburn, MA, United States of America) library quantification kits. The library was sequenced on a NovaSeq PE250 platform at Hangzhou Lianchuan Biotechnology Co., Ltd.

### Statistical Analysis

All the data were organized by using Microsoft Excel 2010. The data on soil nutrients and enzyme activities were statistically analyzed by one-way ANOVA (*P* < 0.05) following the GLM procedures of SPSS statistical software version 23.0 (SPSS Inc., Chicago, IL, United States of America) and Origin 2018 was used for constructing graphs. Alpha and beta diversity values were calculated using QIIME2, and graphs were plotted using R statistical software (version 3.5.2; Venn Diagram, stats, vegan and ade4 package). The species annotation used the feature-classifier plug-in of QIIME2 for sequence comparison. The databases used for comparison were SILVA and NT-16S databases, and the results of SILVA database annotations shall prevail. Redundancy analysis (RDA) was performed to analyze the correlation of the soil bacterial communities with the environmental factors using Canoco software (Version 5.0).

## Results

### Soil Nutrient Content and Enzyme Activities

**Figure 1:**
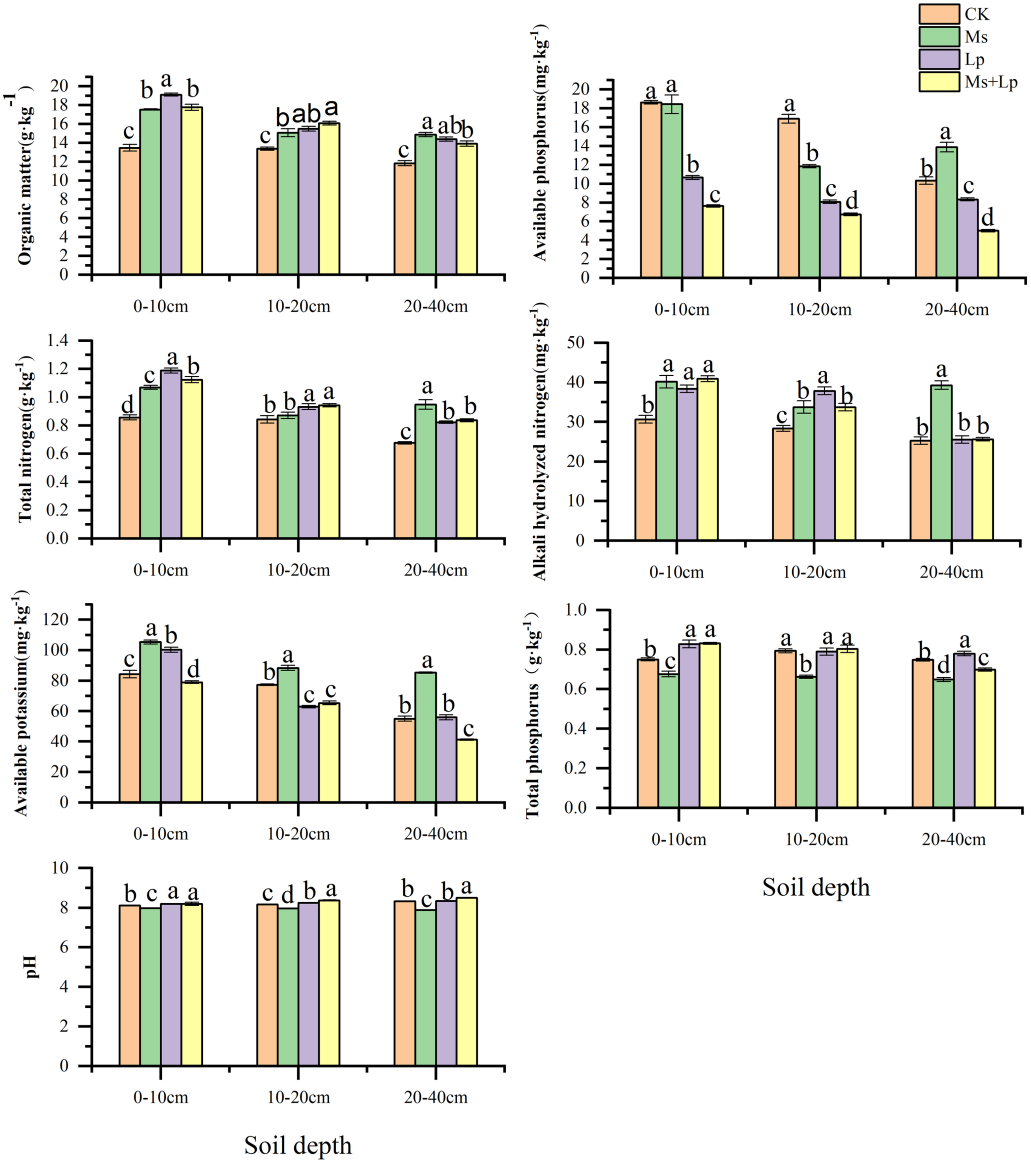
Effects of different grass patterns on soil physical and chemical properties Different lowercase letters in the figure indicate significant (*P* < 0.05).

**Figure 2:**
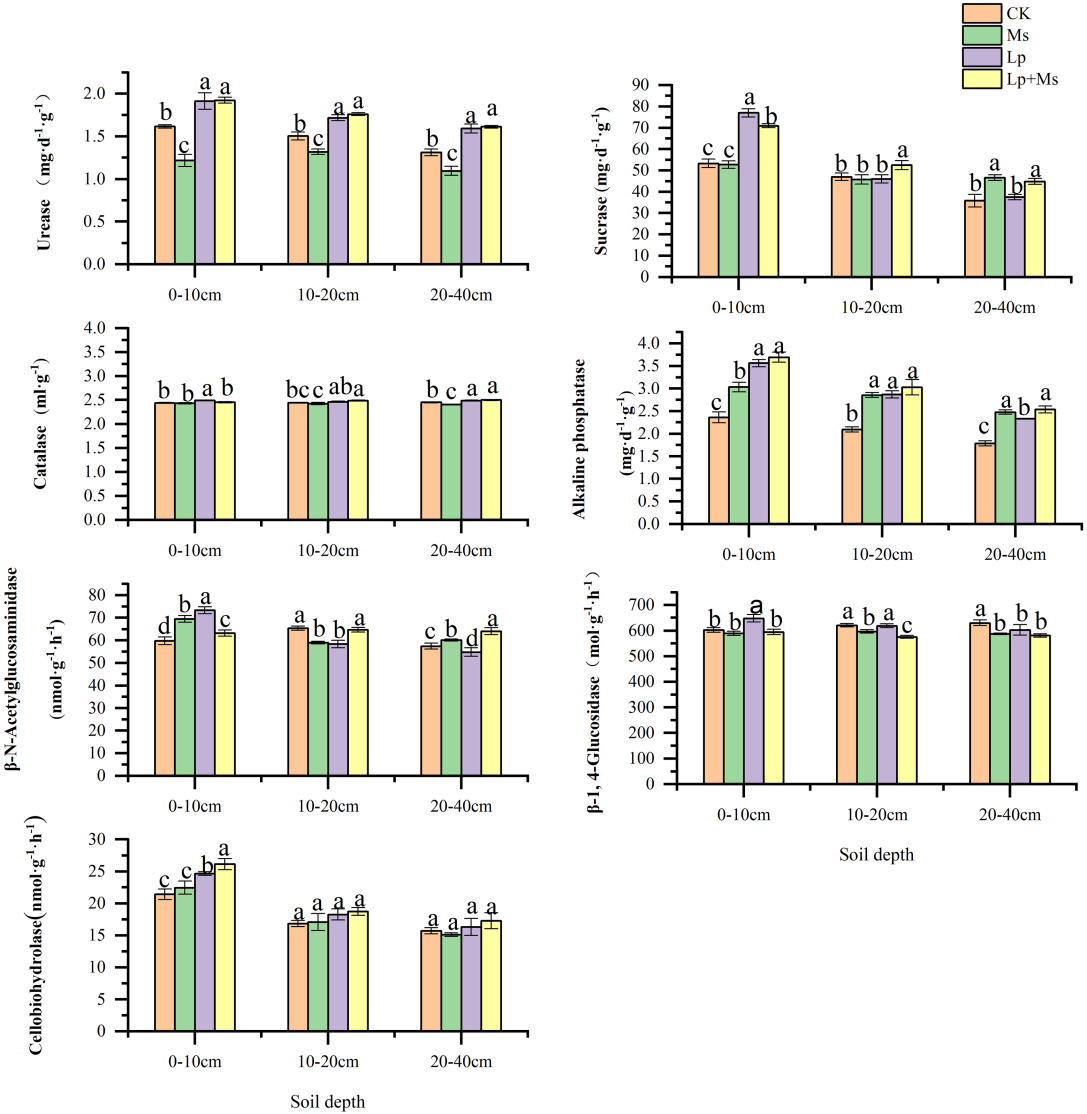
Effects of intercropping grass on enzyme activities in different soil layers. Different lowercase letters in the figure indicate significant (*P* < 0.05).

Compared with CK, intercropping grass could significantly improve soil chemical properties, mainly in the shallow soil layer (0–20 cm), and the effect gradually decreased with the increase of soil depth. From Figure 1, It can be seen that OM, TN and AN had significant effects on the 0–10 cm soil layer (*p* < 0.05), and the increase ranges were 31.76–41.70, 24.85–38.71, and 25.10–33.38%, respectively. In the 10–20 cm soil layer, grass-species intercropping had a significant indigenous effect on OM and AN contents (*P* < 0.05), with a range of 12.69–20.35 and 19.00–33.48%; in 20–40 cm soil layer, the contents of OM and TN in intercropped grass had significant indigenous effects (*P* < 0.05), and the ranges were 17.69–25.74 and 21.54–40.17%, respectively. The effects of different inter-planting treatments on soil enzyme activities are presented in Figure 2. Compared with CK, the Lp + Ms. significantly (*P* < 0.05) increased the activities of URE, SUA, ALP, and CB in the 0–40 cm soil layers by 17.05–22.98%, 11.78–33.10%, 41.98–56.39%, 10.01–21.95%, respectively. Meanwhile, the URE and BG activities decreased in the MS, of which the effect on URE was significant (*P* < 0.05). The treatment increased the ALP activity in the soil, consistent with the change in AP content. The soil URE and ALP activities increased in the Lp, and the effects on URE (14.20–21.38%) and ALP (30.48–50.84%) activities were significant (*P* < 0.05).

### Alpha Diversity of Soil Bacterial Communities

**Figure 3:**
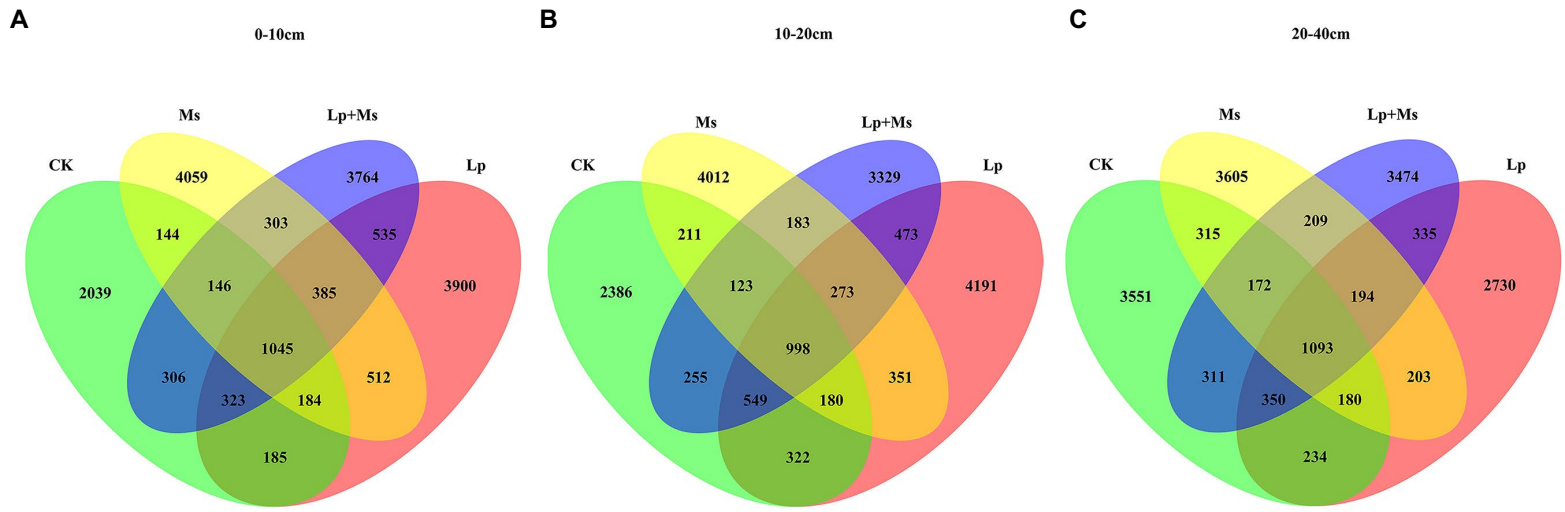
Number of features in different intercropping grass modes: **(A)** 0–10 cm; **(B)** 10–20 cm; and **(C)** 20–40 cm.

Figures 3A,B show the impact of different cover crops on the diversity of soil microbial communities in the 0–20 cm soil layer. The data showed that Lp has the highest population abundance. The number of features was the highest for the Ms., followed by the Lp + Ms. The species richness was also analyzed (Table 1); the different inter-planting treatments showed significant effects on the Chao1 and Shannon diversity indexes of soil bacterial communities, mainly in the 0–10 cm soil layer. Among them, Lp was the best, followed by the Lp + Ms. Both these treatments showed significant (*P* < 0.05) effects compared with CK. In the 10–20 cm soil layer, the indexes of the Lp were better than the other groups, with significantly (*P* < 0.05) higher values than the Lp + Ms. The coverage index was around 98% for all four groups, indicating a high soil bacterial coverage. Overall, perennial ryegrass showed the greatest influence on bacterial diversity in the 0–10 cm soil layer.

**Table 1:** Effects of different interrow grasses on Alpha diversity of soil bacteria.

Soil depth	Items	Group			
		Group CK	Group Lp	Group Ms	Group Lp + Ms
0–10 cm	Shannon index	9.83 ± 0.53^b^	10.57 ± 0.09^a^	10.42 ± 0.06^a^	10.50 ± 0.04^a^
	Simpson index	0.9982 ± 0.00^a^	0.9988 ± 0.00^a^	0.9988 ± 0.00^a^	0.9989 ± 0.00^a^
	Chao1 index	1653.63 ± 594.27^b^	2782.41 ± 201.62^a^	2591.94 ± 170.12^ab^	2650.28 ± 74.63^a^
	Coverage	0.99 ± 0.00	0.97 ± 0.00	0.98 ± 0.00	0.98 ± 0.00
10–20 cm	Shannon index	10.13 ± 0.35^ab^	10.51 ± 0.04^a^	10.33 ± 0.15^ab^	10.34 ± 0.07^b^
	Simpson index	0.9985 ± 0.00^a^	0.9988 ± 0.00^a^	0.9986 ± 0.00^a^	0.9987 ± 0.00^a^
	Chao1 index	2035.29 ± 597.11^ab^	2882.70 ± 191.63^a^	2509.48 ± 258.40^ab^	2431.07 ± 121.42^b^
	Coverage	0.99 ± 0.00^a^	0.97 ± 0.00^a^	0.98 ± 0.00^a^	0.98 ± 0.00^a^
20-40 cm	Shannon index	10.31 ± 0.06^a^	10.15 ± 0.06^b^	10.20 ± 0.20^ab^	10.28 ± 0.08^a^
	Simpson index	0.9987 ± 0.00^a^	0.9985 ± 0.00^a^	0.9985 ± 0.00^a^	0.9986 ± 0.00^a^
	Chao1 index	2449.24 ± 159.51^a^	2149.19 ± 120.43^a^	2248.39 ± 366.54^a^	2408.22 ± 226.19^a^
	Coverage	0.98 ± 0.00^a^	0.98 ± 0.00^a^	0.98 ± 0.00^a^	0.98 ± 0.00^a^

The results are presented as means ± SD. In the same row, values with no letter or the same letter superscripts indicate no significant difference (*P* > 0.05), while with different small letter superscripts indicate significant difference (*P* < 0.05).

### Beta Diversity of Soil Bacterial Communities

**Figure 4:**
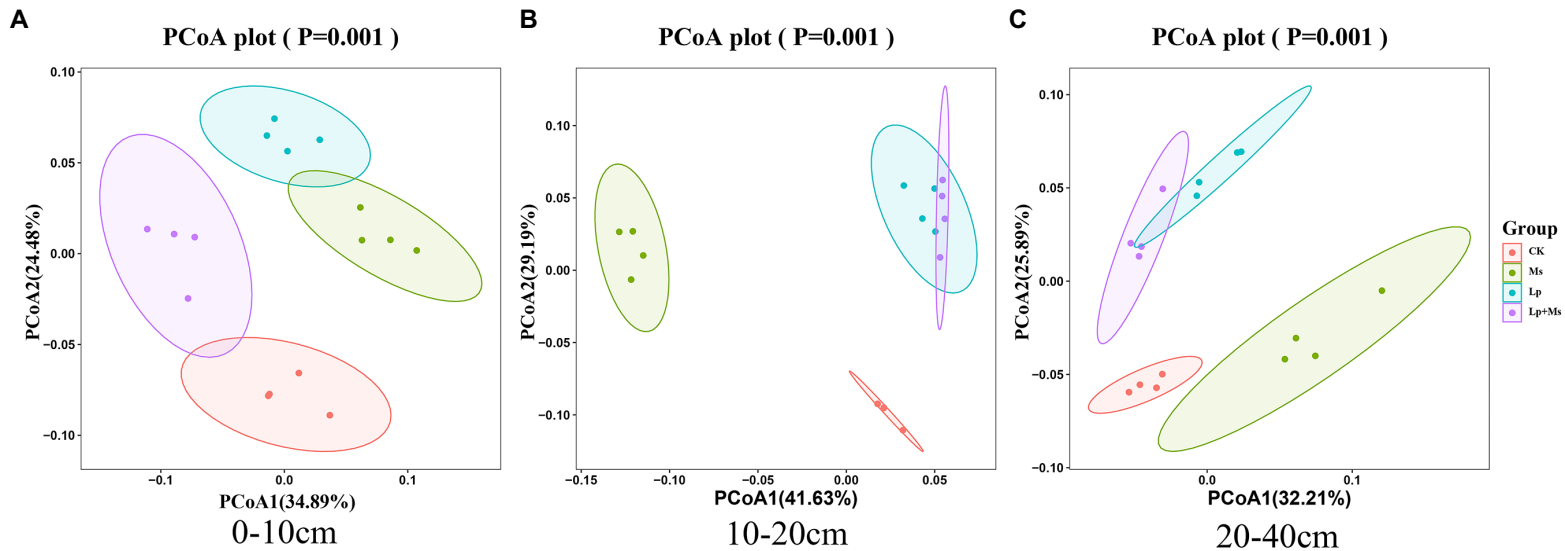
Beta diversity in different soil layers under intercropped grass. **(A)** 0–10 cm; **(B)** 10–20 cm; and **(C)** 20–40 cm represent the different soil layers.

Principal coordinate analysis (PCoA) for different samples based on the Weighted_UniFrac distance matrix is shown in Figure 4. The first principal coordinates (PCoA1) and the second principal coordinates (PCoA2) explained 59.37% (Figure 4A), 70.82% (Figure 4B), and 58.10% (Figure 4C) soil bacterial structure differences in the different soil layers. The orchard’s different cover crops significantly changed the soil bacterial community structure, which was evident in the 0–10 cm (Figure 4A) and 10–40 cm soil layers (Figures 4B,C) of Lp group. The community structure was similar to that of the Lp + Ms., but significantly different from that of the Ms., indicating the influence of Lp.

### Bacterial Community Composition at the Phylum Level

**Figure 5:**
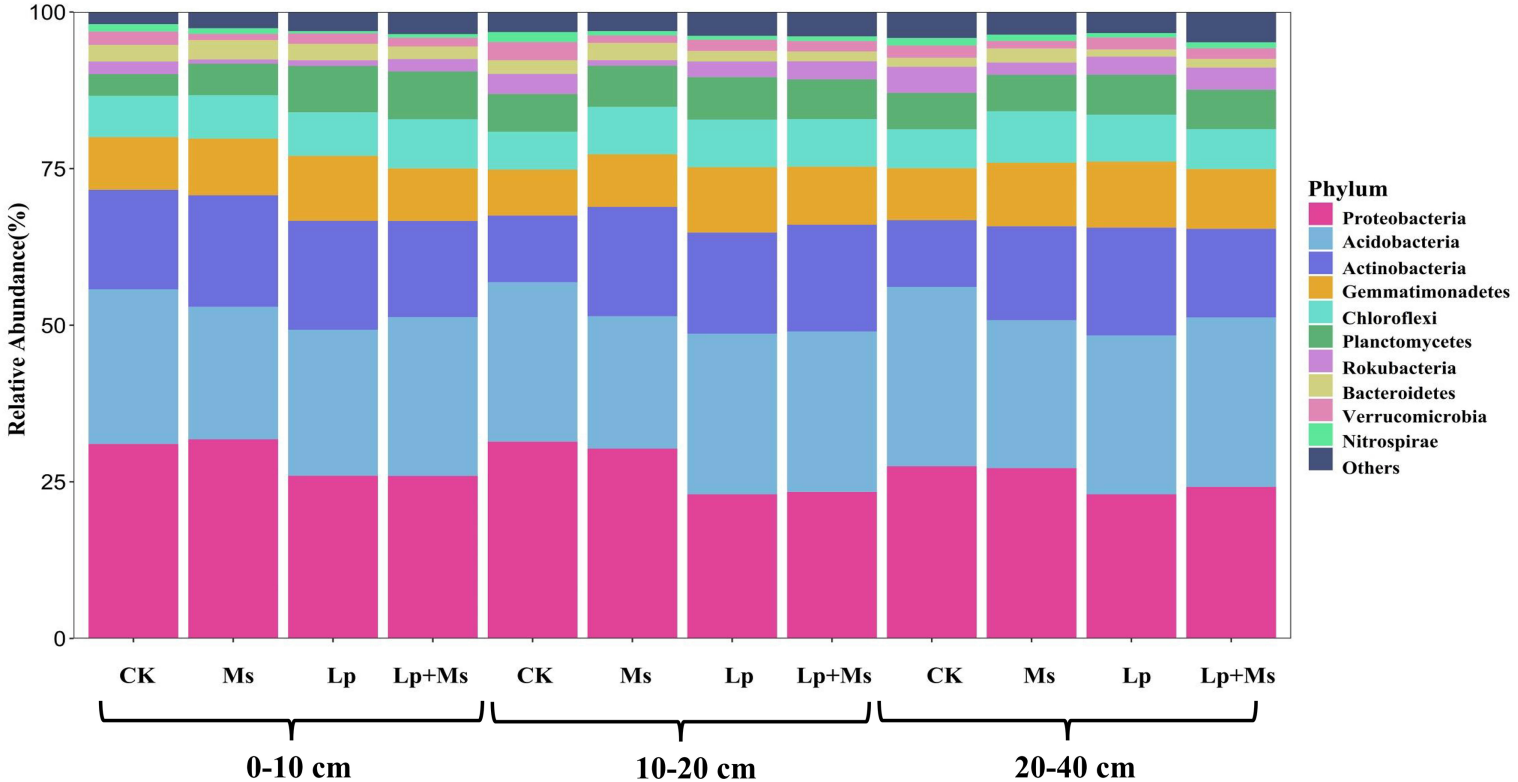
Composition of soil bacterial communities under different grass patterns at phylum and genus levels.

**Figure 6:**
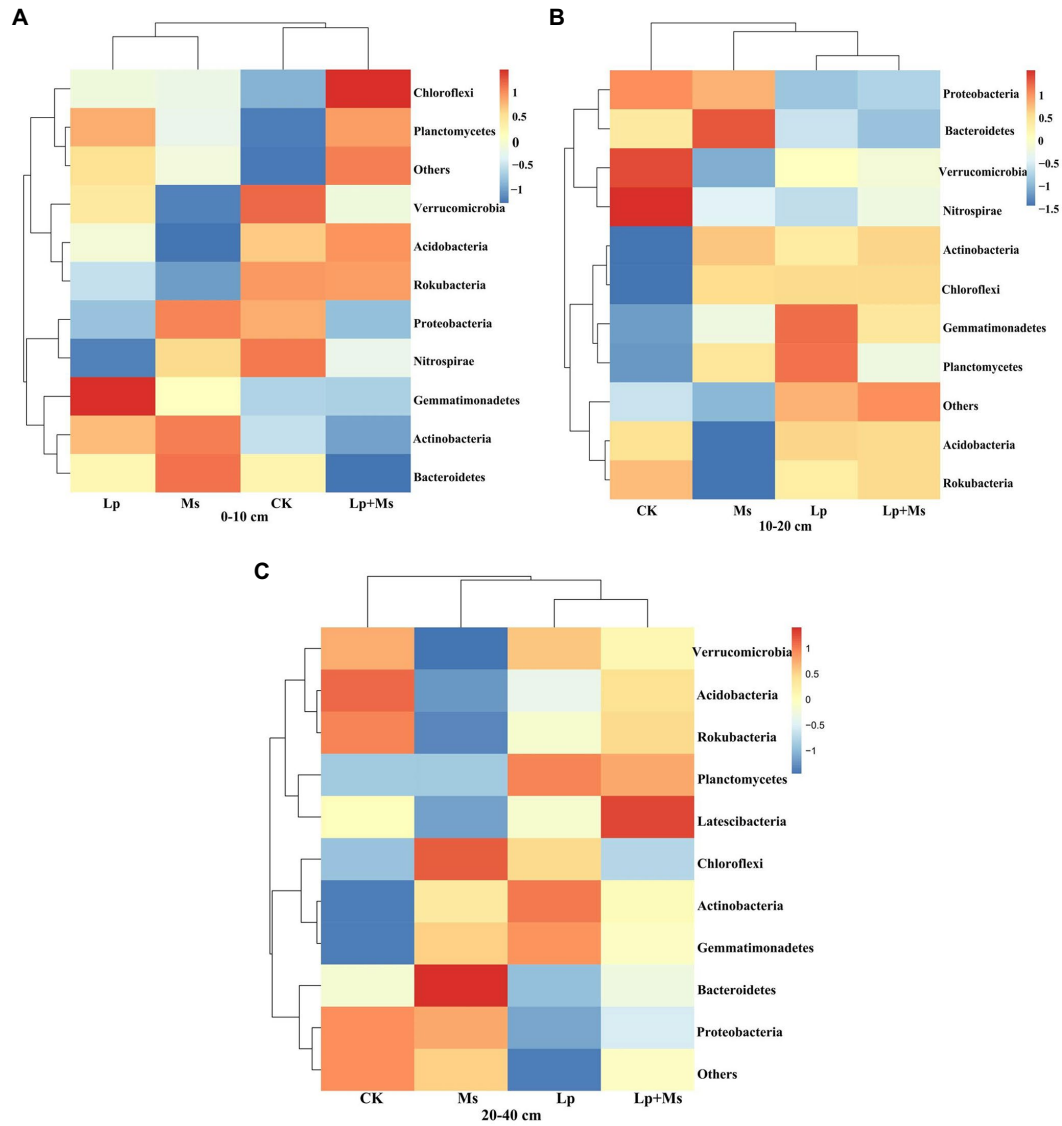
Bacterial population abundance in different soil layers at phylum level: **(A)** 0–10 cm; **(B)** 10–20 cm; and **(C)** 20–40 cm.

From all groups, a total of 31 phyla were identified and classified at the phylum level (Figure 5). The top ten dominant phyla were *Proteobacteria*, *Acidobacteria*, *Actinobacteria*, *Gemmatimonadetes*, *Chloroflexi*, *Planctomycetes*, *Rokubacteria*, *Bacteroidetes*, *Verrucomicrobia*, and *Nitrospirae*. In 0–10 cm soil layer (Figure 6A), *Gemmatimonadetes* (10.37%) and *Planctomycetes* (7.40%) in Lp group were significantly higher than those in CK group (*P* < 0.05). The relative abundance of *Planctomycetes* (7.64%) in the Lp + Ms. group was significantly (*P* < 0.05) higher than that of the CK group, while the relative abundance of *Chloroflexi* (7.84%) was significantly (*P* < 0.05) higher than the other three groups. In the 10–20 cm (Figure 6B), *Verrucomicrobia* and *Nitrospirae* significantly (*P* < 0.05) decreased in the treatment. Compared with CK, the cover crops significantly increased the population of *Actinobacteria* (51.00–64.00%), *Gemmatimonadetes* (14.75–42.49%), and *Chloroflexi* (25.12–25.62%) but significantly (*P* < 0.05) reduced the relative abundance of *Rokubacteria*, *Verrucomicrobia*, and *Nitrospirae* in this soil layer. In the 20–40 cm (Figure 6C), cover crops significantly (*P* < 0.05) increased the relative abundance of *Gemmatimonadetes* (14.92–26.71%) and *Chloroflexi* (2.41–31.99%) while they significantly (*P* < 0.05) reduced the relative abundance of *Rokubacteria* and *Nitrospirae*.

### RDA Analysis Between Species and Environmental Factors

**Figure 7:**
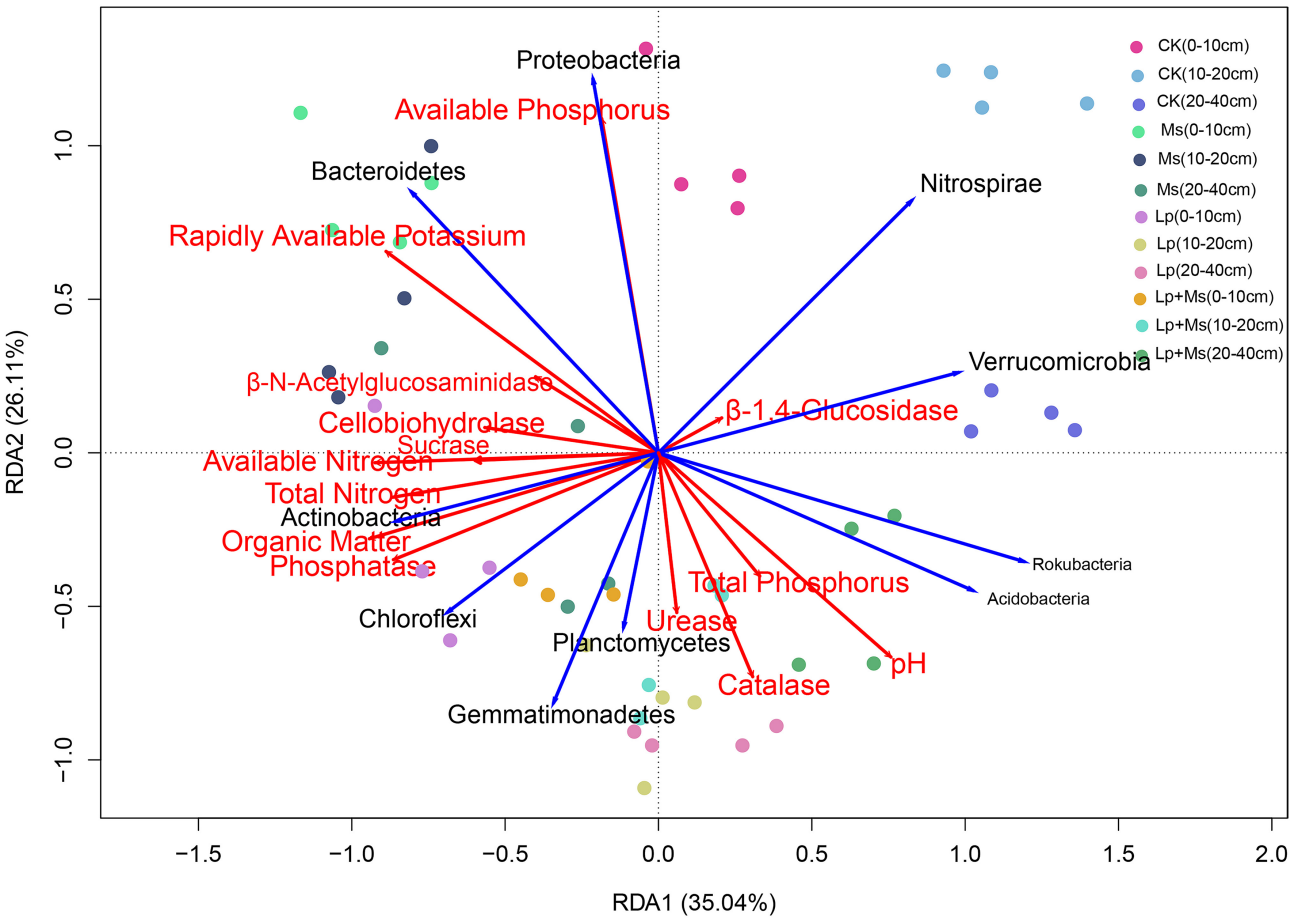
Redundancy analysis between soil nutrients and dominant bacterial communities.

Redundancy analysis (RDA) was used to determine the impact of soil nutrient on the bacterial community and the relationship between samples, enzymatic activity, and bacterial community. As shown in Figure 7, the correlation between the soil nutrient, enzyme activity, and bacterial community. RDA1 and RDA2 explained 35.04 and 26.11% of the total variation. The analysis revealed that the cover crops had obvious effects on soil nutrients, enzyme activities, and dominant bacteria. *Actinobacteria* was positively correlated with ALP, OM, TN, and AN; *Nitrospirae*, *Verrucomicrobia*, *Rokubacteria*, and *Acidobacteria* were negatively correlated with OM, TN, and AN; *Bacteroidetes* was positively correlated with AK and AP and negatively correlated with pH, TP, URE, and CAT.

## Discussion

### Effect of Grass Inter-Planting in Orchard on Soil Nutrients and Enzyme Activities

Soil nutrients directly affect tree growth, fruit yield, and quality. Researchers have established a close correlation between soil fertility and sustainable production in orchards (Wardle et al., 2001; Milgroom et al., 2007). Results from the present study shows that cover crops can significantly improve soil nutrients, which may be related to the accumulation and degradation of plant litter, including the roots. Tagliavini et al. (2007) showed that the grass’s nutrient elements are gradually released into the soil, changing the soil element content. Among the different treatments of this study, inter-planting Lp and Ms between rows in an apple orchard resulted in the most significant effect on OM, TN, and AN contents of the 0–10 cm soil layer, consistent with the reports by Sanchez et al. (2007) and Qian et al. (2015). The OM content of the 0–10 cm soil layer of perennial ryegrass was higher than that of alfalfa, which may be related to the planting age and root distribution (Gong et al., 2018). Meanwhile, alfalfa significantly increased the 20–40 cm soil layer’s AN content, which may be related to the interaction between alfalfa and soil rhizobia for nitrogen fixation. This further increases the nitrogen use efficiency (Kamh et al., 1999; Yao et al., 2005; Hoagland et al., 2008). This is consistent with the study of Hui et al. (2010) on planting white clover and alfalfa between vineyard rows. However, the 0–20 cm soil layer’s AP content was lower than that in the CK group, which indicates that perennial ryegrass consumes more phosphorus and needs phosphorus fertilizers during the early growth stage. However, the content of AP in the 20–40 cm soil layer of the Ms. group was higher than that CK, indicating a restorative increase in the soil AP content in this layer. This effect may be related to the distribution characteristics of alfalfa roots as alfalfa roots are more widely distributed in 20–40 cm soil layer, which is conducive to the return of dead roots and metabolites to soil (Qin et al., 2020).

Soil enzymes are the important components of soil ecosystem with catalytic roles and the metabolic power of soil organisms. They are closely related to soil physical and chemical properties, soil types, fertilization, tillage and other management measures (Badiane et al., 2001). In this study, the inter-planting of perennial ryegrass and alfalfa combination (Lp + Ms) and perennial ryegrass alone (Lp) between the orchards’ rows improved the CBH activity, which are consistent with the findings of Zheng et al. (2018). Compared with CK, the grass combination significantly increased the URE, SUA, and ALP activities in the soil layer analyzed. Alfalfa (Ms) increased the ALP activity while perennial ryegrass (Lp) increased the soil URE, ALP, and CAT activities. These results are consistent with the findings of Zuo et al. (2019) on soil URE, SUA, CAT and ALP activities after grass planting in persimmon orchard in Weibei. In this study, soil enzyme activities were enhanced after grass growing, especially in the shallow soil layer because grass growing in the near surface soil where animal and plant residues and microorganisms are concentrated, strengthening the substrates of enzymes, thus increasing the enzyme activities (Zuo et al., 2019). Additionally, cover crops in the orchard preserve soil moisture to a certain extent, providing a suitable temperature and humidity for soil enzymes (Bradley and Fyles, 1995; Yang and Wang, 2002). Therefore, orchard grass can promote soil enzyme activity and accelerate humus substance metabolism.

### Effect of Cover Crops in an Orchard on Soil Bacterial Community Diversity

As an important biological indicator of soil health, soil bacteria show changes in their community structure and diversity based on agricultural management measures, which affect the nutrients cycle and energy conversion of the agricultural ecosystem (Nair and Ngouajio, 2012; Qiao et al., 2012). Cover crops improve soil structure, regulate soil temperature and humidity, and enhance soil fertility and other ecological factors (Tu et al., 2006; Jumpponen and Brown, 2014). Consequently, the use of ground cover in the orchard will affect the growth, metabolism, and reproduction of soil bacteria, thereby influencing the community structure and diversity. In this study, inter-planting grass in the orchard significantly increased the bacterial diversity and abundance of the soil, especially in shallow layers (0–20 cm). Studies have shown significant changes in soil bacterial community structure and increase in diversity with grass or organic material mulch (manure and crop straw; Tu et al., 2006; Liu et al., 2008). Similarly, grass inter-planting produces plant litter, which accumulates organic matter and thereby increases bacterial biomass. Besides, the increase in the root biomass enhances root secretions which are beneficial to soil microorganisms, improving the bacterial diversity.

Previous studies have shown *Proteobacteria*, *Actinobacteria*, *Acidobacteria*, *Bacteroidetes,* and *Gemmatimonadetes* as the main bacterial phyla (Tripathi et al., 2014; Zhang et al., 2017). Our findings are consistent with these previous reports. In the present study, different inter-planting treatments significantly increased the relative abundance of *Actinobacteria*, *Gemmatimonadetes*, and *Chloroflexi* in soil, consistent with the findings of Qian et al. (2018). Among these abundant bacteria, *Acidobacteria* generally prefers acidic soils (Xiong et al., 2012; Yuan et al., 2014; An et al., 2019; Horton et al., 2019), and its relative abundance in the soil is negatively correlated with pH (Jones et al., 2009; Yang et al., 2019). After grass inter-planting, forage root exudates lower the soil pH (Dong et al., 2015), conducive to the survival of *Acidobacteria. Gemmatimonadetes* are plant growth-promoting bacteria, which interact with plants to carry out biological nitrogen fixation and induce plants to secrete plant hormones (Li and Han, 2015). Researchers have isolated this phylum from various plants (Taghavi et al., 2009), which supports the increase in the phylum’s relative abundance after weeding. Meanwhile, *Chloroflexi* is a phylum that participates in a series of important biogenic elements such as carbon, nitrogen, and sulfur. These biochemical processes promote the utilization of soil nutrients. Results from our present study also portrayed that with the increase in soil depth, the abundance of *Chloroflexi* decreased, which inhibited the growth of other nutrient-rich bacteria. Although *Chloroflexi* has a high demand for soil nitrogen, yet it is unable to fix it (Campos et al., 2016).

The composition of soil bacterial community is closely related to various environmental factors such as soil nutrient content and soil physical and chemical properties (Campos et al., 2016). The RDA explained the correlation between samples and environmental factors. According to the comprehensive analysis of the sample information and environmental factors, pH showed a significant impact on *Acidobacteria*, consistent with the study by Jones et al. (2009). Compared with the control treatment, grass inter-planting had a noticeable effect on the soil physical and chemical properties and enzyme activities. It also impacted *Gemmatimonadetes*, *Chloroflexi*, *Planctomycetes*, and *Bacteroidetes* at the phylum level and *Phingomonas* at the genus level. *Bacteroidetes* are the main mineralizers of organic carbon, and *Planctomycetes* are important for the soil nitrogen cycle (Guo et al., 2015). However, the role of *Chloroflexi* in the soil ecosystem needs to be investigated.

Intercropping grass increased the soil organic matter, carbon, and nitrogen contents and altered the soil microbial community structure (Fu et al., 2015). It provides a suitable environment for the growth and production of the bacterial community, enhances enzyme activity, and promotes the activity of the bacterial population in nutrient cycling, organic matter decomposition, and energy flow which contributes to the accumulation of organic matter and bioavailability of mineral nutrients. A previous study reported that intercropping grass increased the supply of organic carbon in the soil, which was conducive to the formation of porous soil aggregates, improving soil aeration, and water permeability (Linquist et al., 2012). As a result, it can effectively increase the water retention capacity of orchard soil, accelerate microbial decomposition and consumption of organic carbon, generate more root exudates, activate the mineral elements in the soil, and improve the organic matter content (Hipps and Samuelson, 1991).

## Conclusion

This study suggested that intercropping forages in orchards improved soil nutrients, enzyme activities and bacterial community composition, mainly in the shallow soil layer (0–20 cm). Intercropping perennial ryegrass (Lp) in orchard significantly increased the contents of organic matter, total nitrogen and alkali-hydrolyzable nitrogen in 0–10 cm soil layer. In addition, it enhanced the activities of urease and alkaline phosphatase, and significantly increased the abundance of Actinomycetes, and Chloroflexi. The Redundant analysis (RDA) showed that Actinomycetes were positively correlated with alkaline phosphatase, organic matter and total nitrogen. Also, a positive correlation between Bacteroidetes and alkaline phosphatase and that of Proteobacteria with available phosphorus was evident. Overall, intercropping perennial ryegrass in orchard showed the best effect on soil quality and can be recommended as an appropriate farming practice for apple orchard in Shaanxi China.

## Data Availability Statement

The datasets presented in this study can be found in online repositories. The names of the repository/repositories and accession number(s) can be found in the article/supplementary material.

## Author Contributions

TL, YW, and ML long designed the methodology of this study. MK, XC, and HT conducted the field work and collected the data. TL and YW analyzed the data. TL wrote the manuscript. All authors contributed substantially to the revision. All authors contributed to the article and approved the submitted version.

## Conflict of Interest

The authors declare that the research was conducted in the absence of any commercial or financial relationships that could be construed as a potential conflict of interest.

## Publisher’s Note

All claims expressed in this article are solely those of the authors and do not necessarily represent those of their affiliated organizations, or those of the publisher, the editors and the reviewers. Any product that may be evaluated in this article, or claim that may be made by its manufacturer, is not guaranteed or endorsed by the publisher.
